# A Bayesian Model of Sensory Adaptation

**DOI:** 10.1371/journal.pone.0019377

**Published:** 2011-04-25

**Authors:** Yoshiyuki Sato, Kazuyuki Aihara

**Affiliations:** 1 Graduate School of Information Systems, The University of Electro-Communications, Tokyo, Japan; 2 Institute of Industrial Science, The University of Tokyo, Tokyo, Japan; Duke University, United States of America

## Abstract

Recent studies reported two opposite types of adaptation in temporal perception. Here, we propose a Bayesian model of sensory adaptation that exhibits both types of adaptation. We regard adaptation as the adaptive updating of estimations of time-evolving variables, which determine the mean value of the likelihood function and that of the prior distribution in a Bayesian model of temporal perception. On the basis of certain assumptions, we can analytically determine the mean behavior in our model and identify the parameters that determine the type of adaptation that actually occurs. The results of our model suggest that we can control the type of adaptation by controlling the statistical properties of the stimuli presented.

## Introduction

### Perception as Bayesian inference

The perception of incomplete information occurs commonly in daily life. For example, objects are often partially hidden from view, requiring estimation of occluded components. Further, certain aspects of our surroundings cannot be predicted. This is true even for the nervous systems within the human body. For example, generation of receptor noise and firing of neurons cannot be predicted. Thus, it is necessary for a person to develop ways to deal with such unknown aspects to gain accurate perceptions of the world.

A number of studies have shown that some aspects of human perceptual and motor systems can be explained by optimal Bayesian observer models that deal optimally with uncertainty (for a review, see [1]). These studies have typically compared the performance of human subjects with that of optimal Bayesian observers, and generally reported that they are approximately identical. That is, human perception has been found to be optimal in this sense.

Adaptation is an important aspect of human perception and action. The external environment is constantly changing, and the human body undergoes continuous changes as a result of injury, growth, and aging. Thus, our perceptual and motor systems must adapt to such changes to accurately perceive and interact with the external world. Adaptation is important not only as a subject of study in itself, but also because it can be used to deduce the mechanisms underlying perceptual and motor systems in psychophysical and brain imaging experiments [2]–[4]. Such experiments are important for investigating the human brain, which, due to ethical issues, cannot be directly examined using other techniques common in neuroscience, such as invasive electrophysiological methods.

Bayesian models of perception raise the possibility that adaptation itself is a result of inferences drawn from changes in the inherent statistical properties of our surroundings and our bodies. Indeed, some researchers have modeled the adaptation of perceptual and motor systems using Bayesian inference [5]–[7], successfully explaining experimental results.

### Two types of adaptation

In this paper, we focus on perceptual adaptation to two temporally separated events, a phenomenon that is found across a broad range of human perceptual modalities [8]–[14].

Previous studies have revealed the existence of two opposite types of temporal adaptation. When audiovisual stimuli separated by fixed temporal intervals are repeatedly presented to subjects, they perceive the stimuli to be simultaneous [8], [13]. This phenomenon is known as “lag adaptation.” The same basic type of adaptation has been found in many areas of psychophysics [10]–[12]. A recent study reported, on the other hand, a type of adaptation that acts oppositely to lag adaptation [9]. The experimenters presented two tactile stimuli to each hand of a subject [9]. Then, it was observed that the adaptation effect occurred in the opposite direction relative to the effect observed in lag adaptation. That is, the participants were less likely to perceive simultaneity for repeatedly presented stimuli, and were more likely to judge stimuli with a reverse temporal order as simultaneous. The authors referred to this type of adaptation as “Bayesian calibration [9].” Although it is clear that there are at least two different types of adaptation, the question of why these different types exist and factors determining which type of adaptation is induced remain unclear.

**Figure 1:**
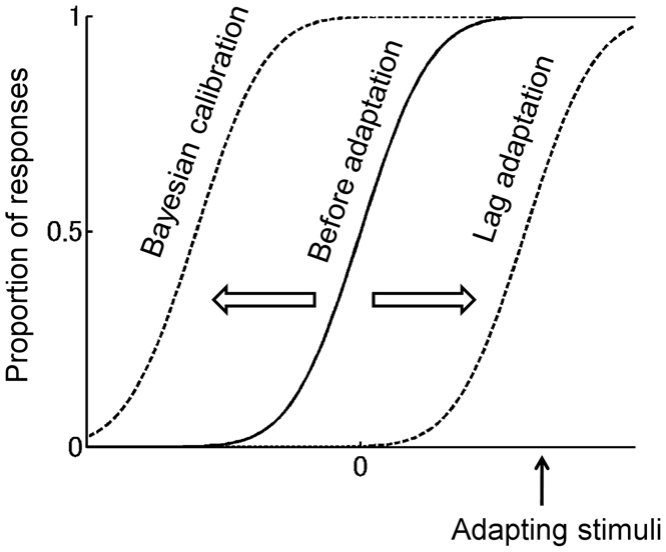
Two types of adaptation effects on a psychometric function. The solid line represents a psychometric function before adaptation, and the other two dotted lines represent psychometric functions after two types of adaptation.

To clarify the characteristics of the two types of adaptation, let us consider a situation in which a pair of stimuli, separated by a time interval, is presented to a subject; the subject's task is to judge which stimulus was presented first. If we plot the percentage of “stimulus 1 was the first” responses for various test temporal intervals, we obtain the psychometric function as shown by the solid line in Figure 1. The center of the psychometric function represents the temporal interval at which the subject judges the pair of stimuli as being simultaneous. During an adaptation period, stimuli with a constant time interval are repeatedly presented. The adaptation stimuli during the adaptation period are not necessarily identical: they may be drawn from a probability distribution. After the adaptation period, we measure the psychometric function again. The type of adaptation is indicated by the difference between the center of the psychometric function plotted before the adaptation period and that drawn after the adaptation period. If the center is shifted toward the adaptation stimuli, the resulting adaptation is of the lag adaptation type, and if it is shifted away from the adapting stimuli, the adaptation is of the Bayesian calibration type (see Figure 1).

### Adaptation in a Bayesian model of perception

In a Bayesian model of perception, it is assumed that the quantity to be perceived (in the example above, a temporal interval between stimuli) cannot be directly observed and that only noisy information can be obtained. This noise might result from noise in sensory organs, uncertainty in firing of neurons, or other factors. If the observer has some knowledge about the quantity, it is a good strategy to use that knowledge in the estimation. Bayesian inference enables the observer to estimate the quantity by combining information gained by observation with prior knowledge. Let *x* denote the temporal interval between stimuli that are presented to the subject. Let *y* denote an observable noisy quantity that is stochastically determined by *x* according to a conditional probability *P*(*y*|*x*). In Bayesian inference, the observer makes an estimation on the basis of the posterior probability of *x* given *y*, i.e., *P*(*x*|*y*). From Bayes' theorem, it follows that
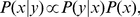
(1)where *P*(*y*|*x*) is the likelihood function that represents the noise distribution and *P*(*x*) is the prior probability distribution of *x* that represents prior knowledge about *x*. Thus, Bayesian inference involves two factors: namely, the likelihood function and the prior probability distribution.

In an earlier study [15], we showed that the ventriloquism aftereffect — the lag-adaptation-type phenomenon observed in audiovisual spatial adaptation — can be explained by adaptive learning of the likelihood function. On the other hand, in [9], it was shown that Bayesian calibration could be explained by assuming that the participants had learned the prior distribution of stimulus timing. Thus, the two types of adaptation are complementary in their phenomenological characteristics and from the viewpoint of Bayesian modeling.

In another study, we extended our model of adaptation to include both types of adaptation and investigated the parameters governing them, and discussed the parameters determining which type of adaptation occurs [16]. However, the physical or physiological meaning of the model parameters remains unclear.

In the present study, we propose a Bayesian model of sensory adaptation. By analyzing the model, we sought to identify the parameters that determine the type of adaptation. Our model suggests that the statistical properties of the presented stimuli affect the type of adaptation; thus, it might be possible to control the type of adaptation that is induced in experiments.

Our aim here was not to quantitatively reproduce the results in the experimental literature, but to provide novel insights about why two types of sensory adaptation exist, and the factors determining the type of adaptation that actually occurs. In addition, we sought to propose an experimental paradigm that could lead to deeper understanding of adaptation.

Here, we consider lag adaptation to represent learning of the likelihood function, and Bayesian calibration to represent learning of the prior distribution. Because both types of adaptation in our model are the result of Bayesian inference and adaptation to temporal lag, the above names of the adaptation types might cause confusion. Therefore, in the following, we refer to lag adaptation as “Type A” adaptation, and Bayesian calibration as “Type B” adaptation.

## Methods

In this section, we first formalize adaptation as the adaptive updating of parameters in the likelihood function and the prior distribution in a Bayesian model of timing perception. Next, we derive updating rules of estimated parameters from the model. Finally, we analytically obtain the center point of the psychometric function after adaptation, and deduce the factors determining the type of adaptation.

### Bayesian model of adaptation

It is common to assume a Gaussian noise distribution. In addition, we introduce a shift of the mean value of the probability distribution [16]. We assume that both the noise distribution (the likelihood function) and the prior distribution of *x* are Gaussian with shifted mean values as follows:
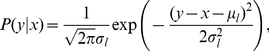
(2)

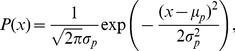
(3)where *σ_l_* and *σ_p_* are standard deviations of the noise distribution and the prior distribution, respectively, and *µ_l_* and *µ_p_* are mean values of the distributions, respectively.

We assume that mean values *µ_l_* and *µ_p_* can change because of certain factors, and we interpret adaptation as the updating of the observer's estimation of *µ_l_* and *µ_p_*. The change in *µ_p_* might result from a change in the statistical properties of the external world, while a change in *µ_l_* may result from changes in sensory organs due to injury, growth, aging, changes in the neuronal encoding of stimuli, or other factors. We discuss this point in detail in the Discussion section.

**Figure 2:**
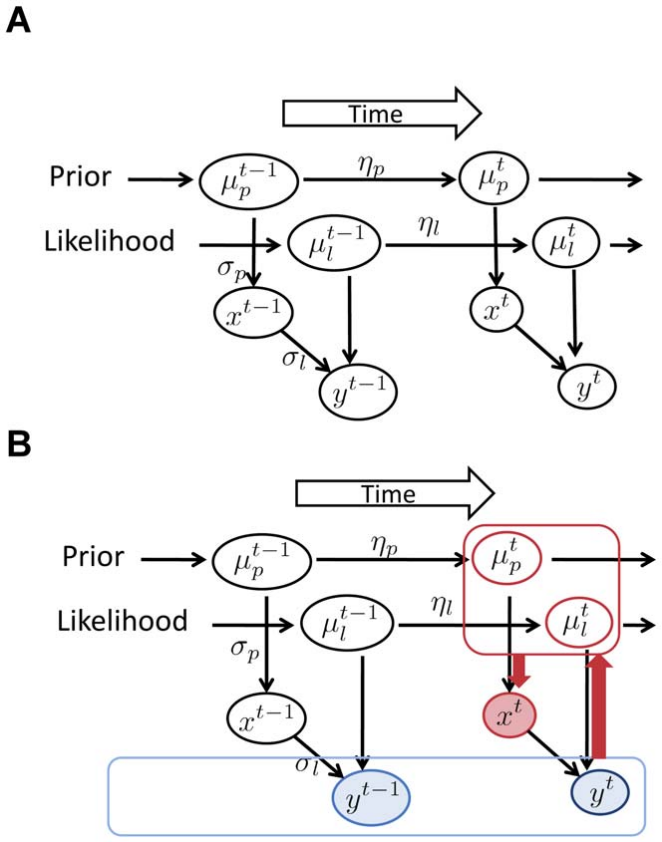
Schematic diagram of our Bayesian model. A: Physical relations of parameters. Values of variables in circles with superscript *t* are stochastically determined at time *t*. The arrows represent causal relations between variables. B: The process of estimation after the observer observes *y^t^* at time *t*. Variables *y* up to time *t* (blue color) are directly observed, and others are not observable. The observer's task is to infer *x^t^*. For the observer to infer *x^t^*, it must also estimate 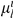 and 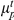.

On the basis of these assumptions, we propose the Bayesian model of adaptation shown in Figure 2. Because the parameter values change from time to time, we denote their values at time *t* with the superscript *t*. Time refers to trials in actual experiments. Figure 2A shows the physical dependence of each model parameter on other parameters. The arrows in Figure 2A show the causal relations between the parameters. Figure 2B shows the process of estimation after the observer observes *y^t^* at time *t*. The quantities that the observer can directly observe are *y* up to time *t*. The observer's task at time *t* is to estimate the real time interval *x^t^*. The observer must estimate 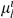 and 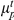 to estimate *x^t^*, and we interpret adaptation as the observer's adaptive learning, i.e., estimations of *µ_l_* and *µ_p_*.

Let us consider an experiment in which the presented stimuli are controlled by an experimenter according to the prior distribution in our Bayesian model, and a subject perceives the stimuli. Here, to make our model analytically tractable, we assume that 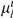 and 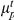 are Gaussian processes; that is, they are determined stochastically by adding Gaussian noise to their previous values. Thus, we assume that
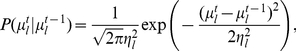
(4)

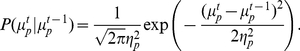
(5)


The parameters *η_l_* and *η_p_* represent the standard deviations of the time evolution of *µ_l_* and *µ_p_*, respectively. We assume that the variance parameters such as *σ_l_*, *σ_p_*, *η_l_*, and *η_p_* are unchanged and that the observer knows them. The parameter 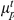 represents the expected value of the presented stimulus at time *t*. The true 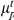 might change from time to time, but its expected value at an arbitrary time *t* is its initial value. The parameter 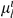 represents the expected value of the sensory noise at time *t*, which the experimenter may not be able to directly observe. It is possible that many of the previous experimental studies on sensory adaptation satisfy these assumptions.

Mathematically, the observer's task is to maximize the posterior probability 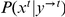, where 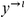 represents the set of all 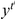 from time 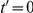 to time 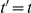. In calculating the estimation of *x^t^*, we need to specify the posterior probability distribution of 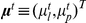 (see Appendix S1 for more detailed mathematical description).

If we assume that 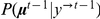 is a normal distribution, then 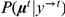 is also a normal distribution, and the mean of 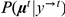 can be interpreted as the observer's estimation of 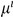. Thus, we assume that
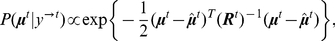
(6)where 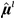 represents the observer's estimation of 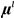 and 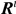 is the covariance matrix of the posterior distribution 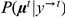 at time *t*.

We can show that the observer's task does not involve the calculation of 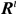 itself, but only 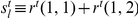 and 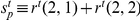, where 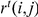
represents the (*i,j*)th component of 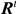. Therefore, we are not concerned about 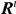 itself, but only about 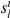 and 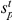 hereafter.

### Assumption of initial convergence

We consider adaptation to be the estimation of the adaptation parameters *µ_l_* and *µ_p_*, which change from time to time. Such changes take place in our daily life. Because the update rule of 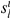 and 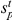
does not depend on the observed or estimated timing of the stimuli (Appendix S1), it might be possible that 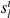 and 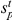 have already been updated a sufficient number of times before the experiment. In addition, it might be possible to design an experiment in which 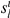 and 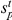 are updated a sufficient number of times. Therefore, we assume that 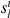 and 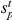 have converged before the experiment.

### Psychometric function

We are interested in investigating what determines the type of adaptation, which is characterized by the center point of the psychometric function. We denote the center point of the psychometric function at time *t* as 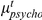.

In practice, to determine 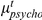 experimentally, test stimuli with various temporal intervals must be presented to a subject, and such stimuli must lead to a change in 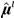 if presented too many times. However, theoretically, we can calculate 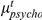 at all times by considering the subject's psychometric function assuming that 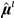 is fixed.

In our model analysis, we determine 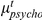 as the time interval where the average estimation of *x^t^* by the observer is 0. Although we can derive the center of the psychometric function more formally [9], [16], this simplified calculation is sufficient for our purpose.

### Analysis of model behavior

From the parameter dependence shown in Figure 2 and the assumptions about the shape of probability distributions, we can derive the update rules of the estimations of 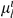 and 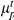
as follows:
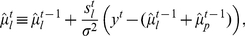
(7)

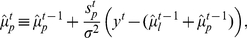
(8)where 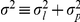. Detailed description of the calculation is provided in Appendix S1.

In practice, although *y* is determined randomly in every trial, we can determine the average behavior of our model by fixing *y* at its expected value 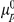 during the adaptation period. First, we investigate the model behavior analytically with this assumption and later validate the analytical result through numerical simulations.

We can solve equations (7) and (8) under the assumption of the initial convergence, and the converged value of 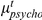 is given by
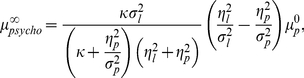
(9)where
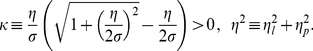
(10)


The type of adaptation is determined by the sign of 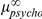 relative to 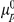, which is the expected value of the presented stimuli. Therefore, equation (9) shows that the type of adaptation is determined by the sign of 
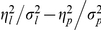
.

## Results

### Numerical simulations

We analytically derived 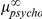 in equation (9) by fixing *y* at its expected value. Here, we conducted numerical simulations to check whether our analytical result matches the simulated behavior.

**Figure 3:**
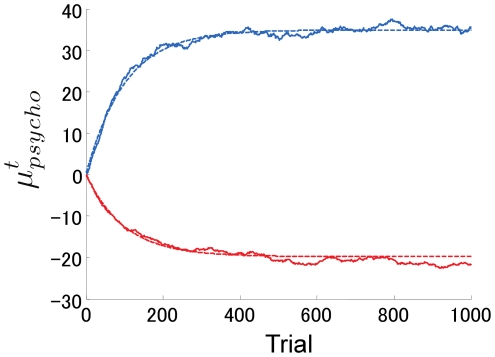
Time course of the center point of the psychometric function. The solid lines show numerical simulation results and the dashed lines show the corresponding analytical results. The two blue lines show the results when 
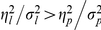
, while the two red lines show the results when 
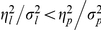
.

Figure 3 shows examples of numerical simulations of 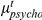 for two sets of parameter values and the corresponding analytical solutions. We assume that the true 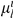 either does not change during this experiment or changes only slightly so that we can neglect its change. Thus, we assume the true 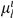 to be 0. We also assume that the observer's initial estimation of 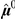 is 0. The parameter values are *x* = 100 ms, *σ_l_* = 50 ms, *η_l_* = 0.5 ms, and *σ_p_* = 0 ms. The solid blue line shows the result for *η_p_* = 0.08 ms, while the solid red line shows the result for *η_p_* = 0.11 ms. The two dashed lines show the corresponding analytical solutions. Figure 3 clearly shows almost perfect agreement between the analytically obtained behavior and the simulated behavior. We confirmed the agreement for all the parameter values we examined. It can also be seen that the type of adaptation is opposite between these two sets of parameter values.

### Model prediction

**Figure 4:**
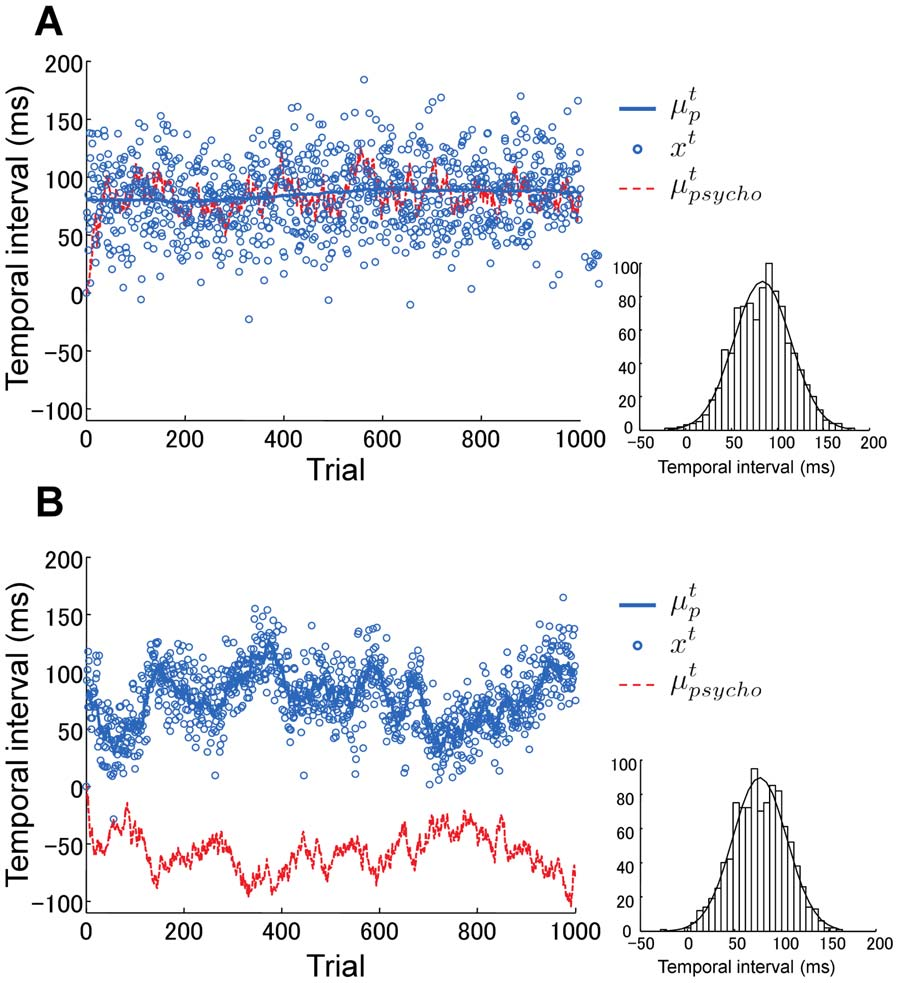
Examples of the time courses of presented stimuli with different statistical properties. A: An example with a small value of 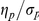. B: An example with a large value of 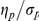. The blue circles represent the temporal intervals of presented stimuli, *x^t^*, and the solid blue lines represent their expected values, 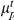. The red dashed lines represent the time courses of the point of simultaneity. Right figures in A and B: the histogram of *x* for each time course.

One important implication made by our model is that the type of adaptation depends on the model parameters, as shown in equation (9). The adaptation is of Type B if 
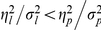
 and of Type A otherwise. The parameters of the prior distribution, *σ_p_* and *η_p_*, describe the statistical properties of the presented stimuli and can be easily controlled in experiments. It means that, by controlling the statistical properties of the presented stimuli, the experimenter might be able to control the type of adaptation. In Figure 4, we show examples of two time series of presented stimuli with small and large values of 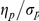, together with the time evolution of 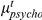. In this figure, we generate *x^t^* and updated 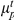 according to equations (3) and (5). The initial value of 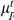 is set to 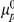 = 80 ms. The parameter values are *σ_p_* = 30 ms and *η_p_* = 0.2 ms in Figure 4A, *σ_p_* = 20 ms and *η_p_* = 3 ms in Figure 4B, and *σ_l_* = 50 ms, *η_l_* = 5 ms in both figures. As can be seen from the two figures, the whole distributions of the stimuli for these sets of parameter values are almost the same. However, our model predicts that the adaptation effects of the two types of stimuli with different temporal structures are completely different.

## Discussion

In this study, we constructed a Bayesian model of sensory adaptation, in which the observer estimates the mean value of the likelihood function and that of the prior probability distribution. We showed that the difference between the ratio of parameters related to the likelihood function and that of the prior distribution, 
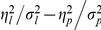
, is essential. Because parameters related to the prior distribution are the statistical properties of the presented stimuli, our model predicted that a stimuli presentation method can dramatically change the type of adaptation.

In most previous studies of temporal perceptual adaptation, adapting stimuli were either fixed (e.g. [8], [13]) or drawn from a Gaussian distribution (e.g. [9]). The latter is clearly the case in Figure 4A. In the former case, the use of fixed stimuli implies that *σ_p_* = *η_p_* = 0 ms, which means that we cannot define its ratio. It is not likely that a human subject can obtain a variance value of exactly zero. Although we must develop a model of the learning of these parameters to fully elucidate this issue, our model suggests that fixing *η_p_* to 0 ms might lead to Type A adaptation. This may explain why Type A adaptation has been observed in most previous experiments. Especially in the case of audiovisual adaptation, the size of adaptation effects was relatively large compared with the temporal interval of adapting stimuli in an experiment in which *σ_p_* had a large value [9], and relatively small in experiments in which *σ_p_* was zero [8], [13], consistently with the results by our model.

It should be noted that we do not claim that the type of adaptation would necessarily differ when we use the sets of stimuli shown in Figures 4A and 4B, because other parameters related to the likelihood functions also affect which type is induced. In fact, in the experiment of Miyazaki et al. [9], adaptation stimuli similar to those shown in Figure 4A were used, and Type B was observed. However, we suggest that, even if the parameters related to the likelihood functions are unknown, a stimulus pattern like that shown in Figure 4A will make the adaptation effect more similar to Type A, while a stimulus pattern like that shown in Figure 4B will make the adaptation effect more similar to Type B. Other parameters should be determined to quantitatively predict the effect of different stimulus presentation methods. The experiment of Miyazaki et al. [9] suggests that the ratio 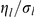
is so small as to be negligible in the tactile system. Therefore, our prediction that the two time courses of stimuli shown in Figures 4A and 4B can lead to different types of adaptation might be better tested in the audiovisual system.

A study by Miyazaki et al. [9] revealed that, even if the same stimulus presentation patterns are used, Type A and Type B adaptation can be observed in the cases of audiovisual sensations and tactile sensations, respectively. In the context of this finding, we now discuss the physical parameter that a subject attempts to estimate in the perception of external stimuli. Let us consider a case in which an external object causes an event in which a pair of audiovisual stimuli are generated, and perceived by the observer. In our model, *x* represents the true time interval between the stimuli that the observer estimates from noisy observations. There are two possible ways to find exactly what *x* represents, i.e., whether it represents the physical time interval between the stimuli when they are delivered to the observer or that when the event occurs. In the case of audiovisual sensations, the former can be large even if the stimuli originate simultaneously, because of (1) the low speed of sound compared with the speed of light and (2) the reflection and diffraction of sound, which result in the sound traveling over a large distance. When the object is still and the surroundings are stable, even if the value of the time difference between the stimuli delivered to the subject is large, the variance of the time difference recorded for many pairs of stimuli would be small. Continuous movements of the object or continuous changes in the surroundings might be a major factor determining the variance of the time difference between the stimuli delivered to the subject. Therefore, it might be the change of the expected value of the time difference that varies from time to time. If we consider *x* as the time interval when the stimuli are presented to the observer, the physical factors that can change the time interval discussed above should be included in the prior distribution of *x* because they are related to the generation of *x*. Thus, in that case, the physical factors should cause a greater increase in *η_p_* than in *σ_p_*. On the other hand, if we consider *x* as the time interval when the event occurs, the physical factors should be considered as the time evolution of *µ_l_*, because it is relevant to the generation of *y* after *x* is determined. This implies that *η_l_* should be large. On the basis of our model, we can say that in a natural environment, the latter interpretation of *x* leads to Type A adaptation, which has been experimentally observed in the case of audiovisual adaptation. Thus, our model suggests that perception as inference is the estimation of the characteristics of the stimuli at their source. This claim is supported by experimental results (e.g., [17]). On the other hand, the origin of a tactile stimulus is a physical touch, and the time interval between the instants at which stimuli are generated is equivalent to the interval between the instants at which the touch stimuli are presented to the subject. Therefore, there is no physical factor that causes a greater increase in *η_l_* relative to that in *σ_l_* in tactile sensations, meaning that tactile adaptation is likely to be of Type B.

Berniker and Kording proposed a motor adaptation model in which internal and external causes of motor errors could be separately estimated [7]. The model we propose here is similar to their model in terms of the notion that the adaptation involves the estimation of different causes: internal and external causes might correspond to *µ_l_* and *µ_p_* in our model, respectively. However, there are some differences between the models. First, in our model, *µ_l_* and *µ_p_* do not necessarily represent internal and external factors as discussed above. Next, in their model, the difference between the estimations of internal and external causes of motor errors is driven by the probability that the external disturbance exists, while in our model, it is the relative relations of variance parameters *σ_l_*, *σ_p_*, *η_l_*, and *η_p_* that drive the difference.

In an earlier study, we constructed a simple integrative model of adaptation [16]. It can be seen that the Bayesian model proposed here has exactly the same mathematical structure as the earlier model if we assume the initial convergence of *s_l_* and *s_p_*. However, the physical or physiological meaning of parameters is much clearer in the presently proposed model, which facilitates control of the types of adaptation.

In this study, for simplicity we assumed that adaptation was the learning of mean parameters 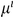, while other parameters related to variances such as *σ_l_*, *σ_p_*, *η_l_*, and *η_p_* were known. Parameters *σ_l_* and *η_l_* might be learned from the subject's past experience. It has been suggested that human subjects can learn the prior distribution from presented stimuli [18], [19]. When we control the prior distribution of the stimuli, we can control their mean values and variances independently. Therefore, it might be possible to set up an experiment in which the variances can be learned before the adaptation of the mean values. However, it is also possible that the learning of the variance variables concurrently occurs with the learning of the mean values. Also, it is not obvious whether the variance parameters related to the likelihood function and those related to the prior distribution can be learned independently. An experimental paradigm in which the parameters of the likelihood function and the prior distribution can be separately measured has been proposed [20], which might enable us to measure the interaction between the learning of the parameters of the likelihood and the prior distribution. The relationship between the learning of the variance parameters and that of the mean parameters, and that between the variance parameters of the likelihood and the prior distribution should be investigated in future.

In equation (9), we showed how the adaptation effect depends on model parameters. This equation is sensitive to changes in the variance parameter values such as *σ_l_*, *σ_p_*, *η_l_*, and *η_p_*, especially when the parameters are set so that the model exhibits Type B adaptation, i.e., 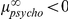. The point of simultaneity, 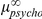, can have a very large negative value, though not a large positive value, depending on the variance parameter values, which might be impossible in human perception. This problem must be discussed together with the learning of the variance parameters *σ_l_*, *σ_p_*, *η_l_*, and *η_p_*. By constructing a model that includes the learning of the variance parameters, we might be able to elucidate whether 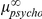
is limited to a plausible value, and how stable the adaptation effect is.

It has been suggested that the causal relationship between stimuli is essential for human perception, action, and adaptation (e.g. [15], [21], [22]). In this paper, we assumed that the observer considers all of the presented stimuli as causally relevant and thus involve them in adaptation. Our model might be extended to explain a broader range of experiments by considering the causal relationship between paired auditory and visual stimuli or between pairs of stimuli.

In the current paper, we investigated a computational model of temporal sensory adaptation. Adaptation is a ubiquitous phenomenon involved in many aspects of human perceptual, cognitive, and motor systems. Therefore, the mathematical structure of our model was formulated in a general form, which can be applied to other aspects of human perception or action only with minor modifications. Adaptation may have different functions in different systems, but it is also possible that it has a common function among different systems in the brain. Thus, our approach, involving abstraction of the computational function of adaptation, provides a plausible approach for investigating the fundamental function of adaptation as a general phenomenon.

## Supporting Information

Appendix S1 **Detailed calculation of the model analysis**
(DOC)
