# Supplementary material for: A Bayesian Model of Sensory Adaptation
Source: PLoS One. 2011 Apr 25;6(4):e19377. doi: 10.1371/journal.pone.0019377 (PMC3081833; doi:10.1371/journal.pone.0019377)
Supplement: Appendix S1 — Detailed calculation of the model analysis (DOC) [file pone.0019377.s001.doc]

# Appendix S1:

# Detailed calculation of the model analysis

From Bayes' theorem and the parameter dependence shown in Figure 2, it follows that

|  | $P(x^{t}\vert y^{\to t})=\int d\mathbf{\mu}^{t}\frac{P(y^{t}\vert x^{t},\mathbf{\mu}^{t})P(x^{t}\vert\mathbf{\mu}^{t})}{P(y^{t}\vert\mathbf{\mu}^{t})}P(\mathbf{\mu}^{t}\vert y^{\to t})$, | (1) |
| --- | --- | --- |

where $\mathbf{\mu}^{t}\equiv(\mu_{l}^{t},\mu_{p}^{t})^{T}$. Therefore, to estimate *x^t^* , we need to know $P(\mathbf{\mu}^{t}|y^{\to t})$, that is, the posterior probability distribution of $\mathbf{\mu}^{t}$.

The posterior probability distribution $P(\mathbf{\mu}^{t}|y^{\to t})$ can be calculated as

|  | $P(\mathbf{\mu}^{t}\vert y^{\to t})\propto\int dxd\mathbf{\mu}^{t-1}P(y^{t}\vert x^{t},\mathbf{\mu}^{t})P(x^{t}\vert\mathbf{\mu}^{t})P(\mathbf{\mu}^{t}\vert\mathbf{\mu}^{t-1})P(\mathbf{\mu}^{t-1}\vert y^{\to t-1})$, | (2) |
| --- | --- | --- |

which can be interpreted as the update rule of the posterior distribution of $\mathbf{\mu}^{t}$.

If we assume that $P(\mathbf{\mu}^{t-1}|y^{\to t-1})$ is a normal distribution, then $P(\mathbf{\mu}^{t}|y^{\to t})$ is also a normal distribution. Furthermore, the mean of $P(\mathbf{\mu}^{t}|y^{\to t})$ can be interpreted as the observer's estimation of $\mathbf{\mu}^{t}$. Thus, we assume that

|  | , | (3) |
| --- | --- | --- |

where $\hat{\mathbf{\mu}}^{t}$ represents the observer's estimation of $\mathbf{\mu}^{t}$, and $\mathbf{R}^{t}$ is the covariance matrix of the posterior distribution $P(\mathbf{\mu}^{t}|y^{\to t})$ at time *t*.

Given this assumption, we can calculate the integrals in equation (2) and rewrite them as update rules for parameters as follows:

|  | $(\mathbf{R}^{t})^{-1}=\mathbf{A}+(\mathbf{R}^{t-1}+\mathbf{H})^{-1}$, | (4) |
| --- | --- | --- |
|  | , | (5) |

where

|  | $\mathbf{A}\equiv\frac{1}{\sigma_{l}^{2}+\sigma_{p}^{2}}\left(\begin{matrix} 1 & 1 \\ 1 & 1 \end{matrix}\right), \mathbf{H}\equiv\left(\begin{matrix} \eta_{l}^{2} & 0 \\ 0 & \eta_{p}^{2} \end{matrix}\right)$. | (6) |
| --- | --- | --- |

We define

|  | $s_{l}^{t}\equiv r^{t}(1, 1)+r^{t}(1, 2)$, | (7) |
| --- | --- | --- |
|  | $s_{p}^{t}\equiv r^{t}(2, 1)+r^{t}(2, 2)$, | (8) |

where $r^{t}(i, j)$ represents the (*i, j*)th component of $\mathbf{R}^{t}$. After some calculation, equation (4) can be rewritten as

|  | $s_{l}^{t}\equiv\frac{\sigma^{2}}{s_{l}^{t-1}+s_{p}^{t-1}+\eta^{2}+\sigma^{2}}(s_{l}^{t-1}+\eta_{l}^{2})$, | (9) |
| --- | --- | --- |
|  | $s_{p}^{t}\equiv\frac{\sigma^{2}}{s_{l}^{t-1}+s_{p}^{t-1}+\eta^{2}+\sigma^{2}}(s_{p}^{t-1}+\eta_{p}^{2})$, | (10) |

where $\eta^{2}\equiv\eta_{l}^{2}+\eta_{p}^{2}$ and $\sigma^{2}\equiv\sigma_{l}^{2}+\sigma_{p}^{2}$. The converged values of $s_{l}^{t}$ and $s_{p}^{t}$ can be calculated as

|  | $s_{l}^{\infty}=\frac{\eta_{l}^{2}}{2\eta^{2}}\left(\sqrt{\eta^{4}+4\sigma^{2}\eta^{2}}-\eta^{2}\right) , s_{p}^{\infty}=\frac{\eta_{p}^{2}}{2\eta^{2}}\left(\sqrt{\eta^{4}+4\sigma^{2}\eta^{2}}-\eta^{2}\right)$. | (11) |
| --- | --- | --- |

Using $s_{l}^{t}$ and $s_{p}^{t}$, equation (5) can be rewritten as

|  | , | (12) |
| --- | --- | --- |
|  | . | (13) |

The estimation of *x^t^* can be calculated from equation (1) as

|  | . | (14) |
| --- | --- | --- |

Equations (9)-(14) show that we do not need to calculate matrix $\mathbf{R}^{t}$ itself, but only $s_{l}^{t}$ and $s_{p}^{t}$. It should be noted that the values of the components of $\mathbf{R}^{t}$ diverge at $t\to\infty$. However, as only $s_{l}^{t}$ and $s_{p}^{t}$ are involved in the observer's task, we are not concerned about the divergence of $\mathbf{R}^{t}$. We assume the initial convergence of $s_{l}^{t}$ and $s_{p}^{t}$ before the experiment as described in the main text.

We can solve equations (12) and (13) under the assumption of the initial convergence of $s_{l}^{t}$ and $s_{p}^{t}$ and by fixing *y* at $\mu_{p}^{0}$ as follows:

|  | $\hat{\mu}_{l}^{t}=\frac{\eta_{l}^{2}}{\eta^{2}}\mu_{p}^{0}\left(1-\left(\frac{\kappa}{\frac{\eta^{2}}{\sigma^{2}}+\kappa}\right)^{t}\right) ,$ | (15) |
| --- | --- | --- |
|  | $\hat{\mu}_{p.}^{t}=\frac{\eta_{p}^{2}}{\eta^{2}}\mu_{p}^{0}\left(1-\left(\frac{\kappa}{\frac{\eta^{2}}{\sigma^{2}}+\kappa}\right)^{t}\right) ,$ | (16) |

where

|  | $\kappa\equiv\frac{\eta}{\sigma}\left(\sqrt{1+\left(\frac{\eta}{2\sigma}\right)^{2}}-\frac{\eta}{2\sigma}\right)>0.$ | (17) |
| --- | --- | --- |

From equations (11), (14), (15), and (16), the converged value of $\mu_{psycho}^{t}$ can be calculated as

|  | $\mu_{psycho}^{\infty}=\frac{\kappa\sigma_{l}^{2}}{\left(\kappa+\frac{\eta_{p}^{2}}{\sigma_{p}^{2}}\right)\left(\eta_{l}^{2}+\eta_{p}^{2}\right)}\left(\frac{\eta_{l}^{2}}{\sigma_{l}^{2}}-\frac{\eta_{p}^{2}}{\sigma_{p}^{2}}\right)\mu_{p}^{0}$. | (18) |
| --- | --- | --- |
